# Predicting contrast-induced nephropathy after CT pulmonary angiography in the critically ill: a retrospective cohort study

**DOI:** 10.1186/s40560-018-0274-z

**Published:** 2018-01-19

**Authors:** Kwok M. Ho, Yusrah Harahsheh

**Affiliations:** 1 Department of Intensive Care Medicine, Royal Perth Hospital, 4th Floor, North Block, Wellington Street, Perth, Western Australia 6000 Australia; 2 School of Population and Global Health, University of Western Australia, Perth, Western Australia Australia; 3 School of Medicine and Pharmacology, University of Western Australia, Perth, Western Australia Australia; 4 School of Veterinary and Life Sciences, Murdoch University, Perth, Western Australia Australia

**Keywords:** Acute kidney injury, Complications, Contrast, Prediction, Venous thromboembolism

## Abstract

**Background:**

It is uncertain whether we can predict contrast-induced nephropathy (CIN) after CT pulmonary angiography (CTPA). This study compared the ability of a validated CIN prediction score with the Pulmonary Embolism Severity Index (PESI) in predicting CIN after CTPA.

**Methods:**

This cohort study involved critically ill adult patients who required a CTPA to exclude acute pulmonary embolism (PE). Patients with end-stage renal failure requiring dialysis were excluded. CIN was defined as an elevation in plasma creatinine concentrations > 44.2μmol/l (or 0.5 mg/dl) within 48 h after CTPA.

**Results:**

Of the 137 patients included, 77 (51%) were hypotensive, 54 (39%) required inotropic support, and 68 (50%) were mechanically ventilated prior to the CTPA. Acute PE was confirmed in 21 patients (15%) with 14 (10%) being bilateral. CIN occurred in 56 patients (41%) with 35 (26%) required dialysis subsequent to CTPA. The CIN prediction score had a good ability to discriminate between patients with and without developing CIN (Area under the receiver-operating-characteristic (AUROC) curve 0.864, 95% confidence interval [CI] 0.795–0.916) and requiring subsequent dialysis (AUROC 0.897, 95% CI 0.833–0.942) and was better than the PESI in predicting both outcomes (AUROC 0.731, 95% CI 0.649–0.804 and 0.775, 95% CI 0.696–0.842, respectively). A CIN risk score > 10 and 12 had an 82.1 and 85.7% sensitivity and 81.5 and 78.4% specificity to predict subsequent CIN and dialysis, respectively. The CIN prediction model tended to underestimate the observed risks of dialysis, but this was improved after recalibrating the slope and intercept of the original prediction equation.

**Conclusions:**

The CIN prediction score had a good ability to discriminate between critically ill patients with and without developing CIN after CTPA. Used together for critically ill patients with suspected acute PE, the CIN prediction score and PESI may be useful to inform clinicians when the benefits of a CTPA scan will outweigh its potential harms.

**Electronic supplementary material:**

The online version of this article (10.1186/s40560-018-0274-z) contains supplementary material, which is available to authorized users.

## Background

Venous thromboembolism (VTE), including deep vein thrombosis (DVT) and pulmonary embolism (PE), is one of the most preventable causes of death and morbidity in hospitalised patients [1, 2]. The incidence of asymptomatic VTE, including PE, in critically ill or injured patients is very high despite anticoagulant prophylaxis [3]. In one cohort study, up to 10% of the patients already had unsuspected DVT at the time of ICU admission [4] and PE accounted for about 1% of all emergency intensive care unit (ICU) admissions [5].

For patients presenting with non-specific symptoms and signs of PE, including chest pain, respiratory failure, or hypotension, a computed tomography pulmonary angiography (CTPA) is often needed to confirm or exclude a life-threatening acute PE. In addition to a small risk of anaphylaxis, use of radiocontrast can induce acute kidney injury or contrast-induced nephropathy (CIN), especially in patients with pre-existing renal impairment. Although some retrospective observational studies have suggested that CIN may have been overemphasised or may not even exist in patients without multiple risk factors for CIN [6, 7], recent randomised controlled trials did demonstrate that risk of CIN in patients at extreme risk for CIN can be attenuated with aggressive interventions [8, 9]. While most patients who develop CIN will not require dialysis and will recover without permanent complications, there is an increasing evidence to suggest that CIN can induce long-term renal damage and mortality in high-risk patients. In the study by Mehran et al., the risks of requiring dialysis for CIN and 1-year mortality were 13 and 33% for those with multiple risk factors for CIN compared to only < 0.5 and 2% for those with the lowest risk of developing CIN, respectively [10]. The clinical significance of CIN was further confirmed by a recent study which showed that nearly one third of the in-hospital mortality after percutaneous coronary intervention was attributable to CIN, and one death could be potentially prevented by preventing nine cases of CIN [11].

The decision to proceed with a CTPA to exclude a life-threatening acute PE in patients with multiple risk factors for CIN is difficult. Theoretically, a CTPA will be justifiable provided its benefits outweigh its harms. In practice, to balance the benefits and risks of a radiocontrast study for critically ill patients is challenging. Firstly, in critically ill patients presenting with symptoms and signs of a life-threatening PE, opportunities to use prophylactic measures against CIN, including aggressive intravenous hydration, are limited [12–15]. Secondly, many risk factors for CIN, including heart failure, hypotension, and increasing age, are also risk factors for mortality in acute PE [10, 16, 17], suggesting that patients who are most at risk of dying from acute PE are, perhaps, also most at risk of developing CIN after a CTPA scan [18, 19].

Data on prediction of CIN are largely derived from cardiology patients who undergo radiological interventions [10]; whether any existing CIN prediction models can reliably predict CIN after a CTPA, especially when applied to the critically ill, remains uncertain. We hypothesised that in critically ill patients with suspected acute PE, their risk of CIN after CTPA can be estimated by the CIN prediction model developed by Mehran et al. [10]. In this study, we assessed the accuracy of the CIN risk prediction score in a cohort of critically ill patients who needed a CTPA to exclude acute PE. In addition, because Pulmonary Embolism Severity Index (PESI) shares a number of predictors with the CIN risk prediction score, we also wanted to compare the ability of these two prediction models in predicting CIN, as well as mortality, in critically ill patients with suspected acute PE requiring a CTPA scan [16].

## Methods

All critically ill adult patients at Royal Perth Hospital intensive care unit who required an urgent CTPA scan to exclude acute PE, between January 2013 and September 2017, were included. The data analysed included age, gender, with factors that were needed to estimate risks of CIN and dialysis, including hypotension, use of intra-aortic balloon pump, congestive heart failure, anaemia, diabetes mellitus, and baseline renal function before the CTPA (http://tools.farmacologiaclinica.info/index.php) [10]. In addition, the information needed to calculate the PESI include underlying cancer, chronic lung disease, hypoxaemia, tachypnoea, hypothermia, altered mental state, and tachycardia were also collected and analysed [16]. In this study, patients with end-stage renal failure requiring long-term dialysis or patients who had a contrast CT chest scan not specifically tailored to look for acute PE were excluded.

Similar to other studies on CIN, CIN was defined as an elevation in plasma creatinine concentrations > 44.2μmol/l (or 0.5 mg/dl) within 48 h after the CTPA in this study—the same time frame used in the original study by Mehran et al. [10] which was not significantly different from the time frame (1 to 4 days) used in most recent interventional and observational studies on CIN [6–9]. As for the requirement for dialysis, this was captured until hospital discharge. The study centre used between 50 and 75 ml of intravenous radiocontrast (either Ultraject Optiray®350: active ingredient ioversol or Omnipaque-350: active ingredient iohexol) for all CTPA scans. All data used in this retrospective cohort study were already collected for administrative purposes, and the clinicians who made the decision to initiate dialysis for the study patients were blinded to the Mehran’s predicted risk of CIN but not the clinical risk factors for CIN. All procedures performed in this study were in accordance with the ethical standards of the institutional and national research committee and with the 1964 Helsinki declaration and its later amendments or comparable ethical standards. Because the study only used clinical data that were already collected for administrative purposes, formal patient consent was considered not necessary, and this study was registered with the Royal Perth Hospital Clinical Safety and Quality Unit (No: 22233) with an intention for peer-review publication.

### Statistical analyses

Categorical and continuous variables with skewed distributions were analysed by Chi-square and Mann Whitney tests, respectively. Area under the receiver-operating-characteristic (AUROC) curve was used to assess whether the existing CIN prediction score and PESI were useful in discriminating between patients with and without CIN and, similarly, for requiring subsequent dialysis. In this study, an AUROC > 0.8, between 0.7 and 0.8, and < 0.7 were defined as good, satisfactory, and unsatisfactory, respectively. Sample size (*N* = 124) was determined by assuming (a) 95% power, (b) the Mehran’s CIN prediction model had an AUROC of 0.8 to predict CIN and the AUROC for PESI was 0.7, and (c) the risk of CIN was 25% after CTPA in the critically ill.

Calibration of the CIN prediction score and PESI was assessed by the Hosmer-Lemeshow Chi-square statistics and by comparing the observed and predicted risks in a calibration plot. If the CIN prediction score was not well-calibrated, an attempt was made to recalibrate the prediction equation’s slope and intercept using logistic regression to see if this could improve the calibration of the score without using different or additional covariates. Missing data for any of the predictors needed for both prediction models were considered as normal to avoid over-inflating the prediction ability of the models. All statistical analyses were performed by SPSS for Windows (version 24.0, IBM, USA) and MedCalc for Windows (version 12.5, Ostend, Belgium), and a two-tailed α-error of < 5% was taken as significant. The TRIPOD Checklist for this study is listed in Additional file 1: Table S1, and non-identifiable data will be available by contacting the corresponding author.

## Results

### Characteristics of the study patients

Of the 141 patients who required a CTPA to exclude acute PE during the study period, 137 patients who did not have end-stage renal failure were included for further analysis. Prior to the CTPA, 77 patients (51%) were hypotensive, 54 (39%) required inotropic support, and 68 (50%) were mechanically ventilated. Acute PE was confirmed in 21 patients (15%) with 14 (10%) being bilateral. Pneumonia was diagnosed in 66 (48%) patients by the CTPA scan. CIN within 48 h after CTPA occurred in 56 patients (41%), with 35 patients (26%) required subsequent dialysis during the same hospital stay. The characteristics and outcomes of the study cohort are described in detail in Table 1, and no patients had missing data on the risk factors needed to estimate the CIN prediction score and PESI, occurrence of CIN, and requirement for dialysis.

**Table 1:** Characteristics and outcomes of the study patients (*N* = 137)

Variables	Median (interquartile range) unless stated otherwise
Age, years	53 (43–69)
Male, no. (%)	97 (70.8)
Comorbidities, no. (%):	
- Diabetes mellitus	41 (30)
- Cancer	28 (20)
- Chronic lung disease	33 (24)
- Congestive heart failure	56 (41)
Baseline organ support therapy, no. (%):	
- Inotropes	54 (39)
- Mechanical ventilation	68 (50)
- Intra-aortic balloon pump	2 (2)
Baseline physical signs, no. (%):	
- Tachycardia (> 110 bpm)	68 (50)
- Hypotension (< 100 mmHg)	70 (51)
- Tachypnoea (> 30 breaths/min)	77 (56)
- Hypothermia (< 36C^°^)	19 (14)
- Altered mental state	38 (28)
- Hypoxaemia (SaO_2_ < 90%)	104 (76)
Haemoglobin concentrations, d/L	110 (89–131)
Haematocrit, %	33 (27–40)
Plasma creatinine conc., μmol/L	89 (64–137)
CIN risk score	9 (4–16)
Pulmonary Embolism Severity Index	144 (99–190)
APACHE II score	23 (17–31)
Outcomes:	
Maximum creatinine conc. within 48 h after CTPA, μmol/L	92 (65–178)
CIN within 48 h of CTPA, no. (%)	56 (41)
Required subsequent dialysis, no. (%)	35 (26)
Acute pulmonary embolism (PE), no. (%)	21 (15)
Bilateral PE, no. (%)	14 (10)
Pneumonia on CTPA, no. (%)	66 (48)
Hospital mortality, no. (%)	69 (50)
Length of ICU stay, days	6 (3–16)
Length of hospital stay, days	13 (7–26)
CIN prediction score > 10: PPV for CIN, %	75.5
CIN prediction score > 10: NPV for CIN, %	86.6
CIN prediction score > 12: PPV for dialysis, %	58.2
CIN prediction score > 12: NPV for dialysis, %	94.0

*APACHE* Acute Physiology and Chronic Health Evaluation, *CTPA* CT pulmonary angiography, *CIN* contrast-induced nephropathy: defined as an elevation in plasma creatinine concentrations > 44.2μmol/l (or 0.5 mg/dl) within 48 h after CTPA, *ICU* intensive care unit, *NPV* negative predictive value, *PPV* positive predictive value, *SaO*_*2*_ arterial oxygen saturation

### Predicting CIN, dialysis, and mortality

**Fig. 1:**
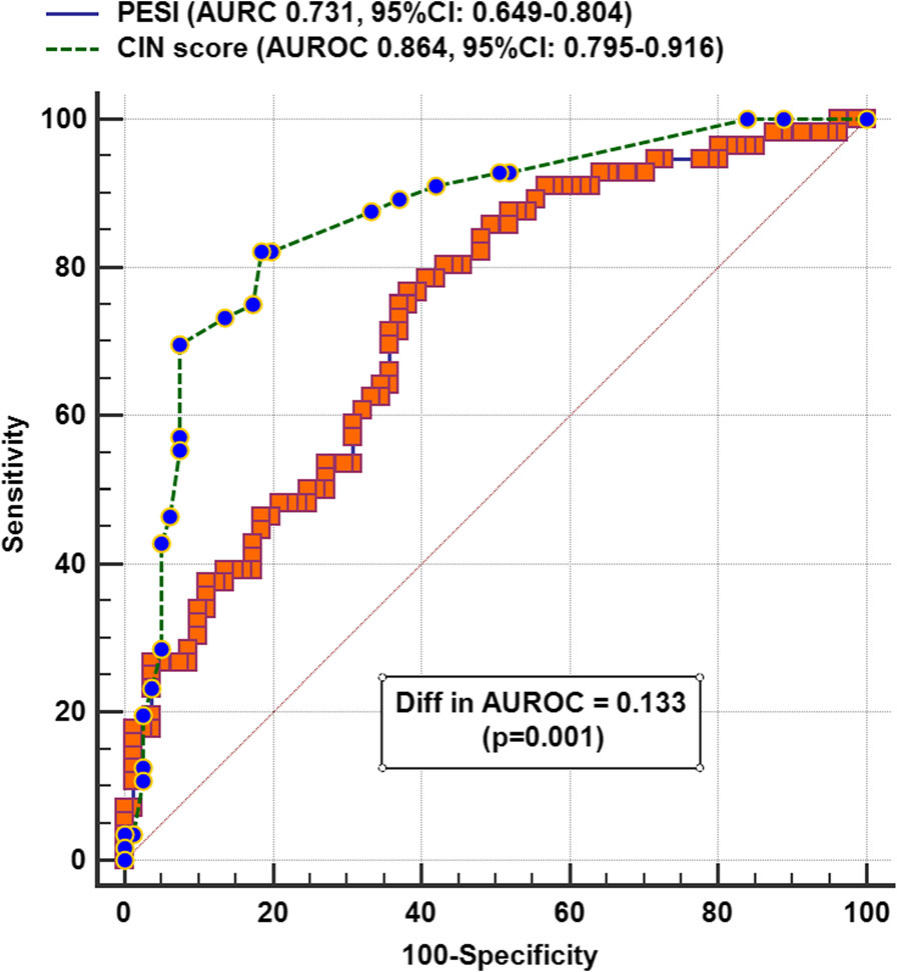
Areas under the receiver-operating-characteristic (AUROC) curve of the Pulmonary Embolism Severity Index (PESI) and contrast-induced nephropathy (CIN) prediction score to predict risk of CIN. CIN was defined as an elevation in plasma creatinine concentrations > 44.2μmol/l (or 0.5 mg/dl) within 48 h after computed tomography pulmonary angiography

**Fig. 2:**
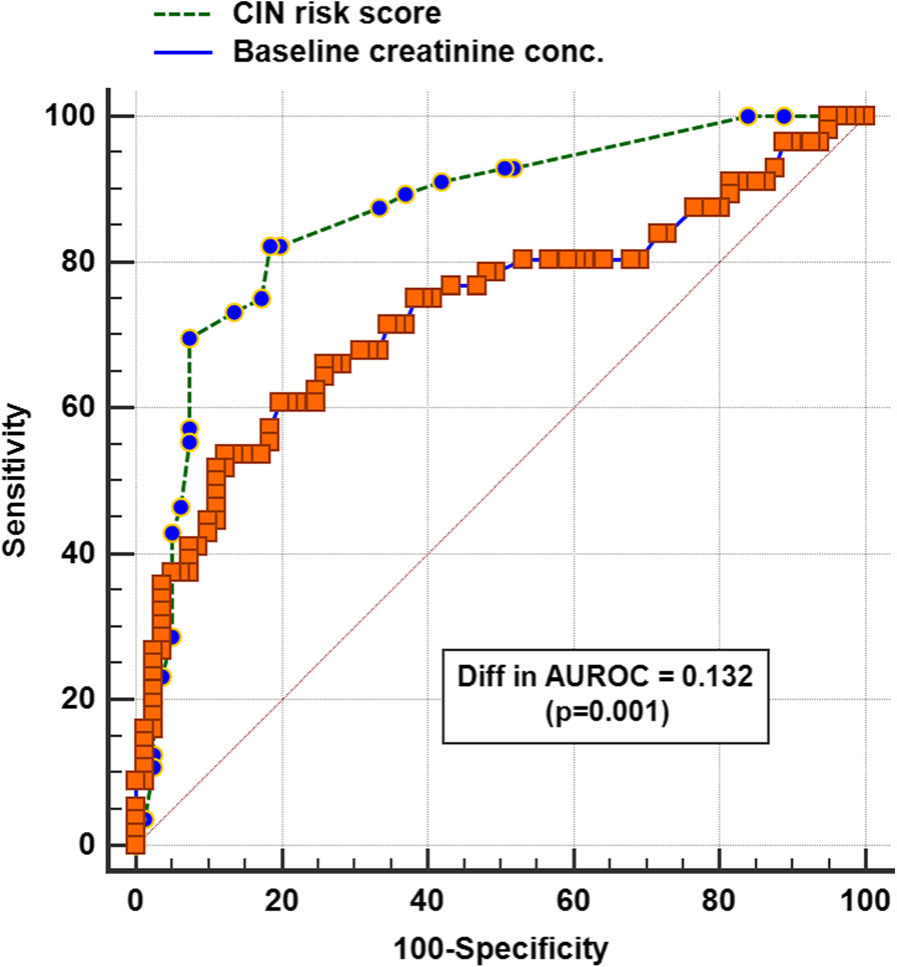
Areas under the receiver-operating-characteristic (AUROC) curve of the contrast-induced nephropathy (CIN) prediction score and baseline plasma creatinine concentration to predict risk of CIN. CIN was defined as an elevation in plasma creatinine concentrations > 44.2μmol/l (or 0.5 mg/dl) within 48 h after computed tomography pulmonary angiography

The CIN prediction score (median 9, interquartile range 4–16) had a good ability to discriminate between patients with and without developing CIN within 48 h after the CTPA (AUROC 0.864, 95% confidence interval [CI] 0.795–0.916), which was better than the PESI (AUROC 0.731, 95% CI 0.649–0.804; difference in AUROC 0.133, *p* = 0.001) (Fig. 1) or using baseline plasma creatinine concentrations alone without the other components of the CIN score (AUROC 0.732, 95% CI 0.641–0.823) (Fig. 2). A CIN risk score > 10 and 12 had an 82.1 and 85.7% sensitivity and 81.5 and 78.4% specificity to predict subsequent CIN (Youden index J 0.64, 95% CI 0.50–0.74) and dialysis (Youden index J 0.64, 95% CI 0.44–0.74), respectively.

**Fig. 3:**
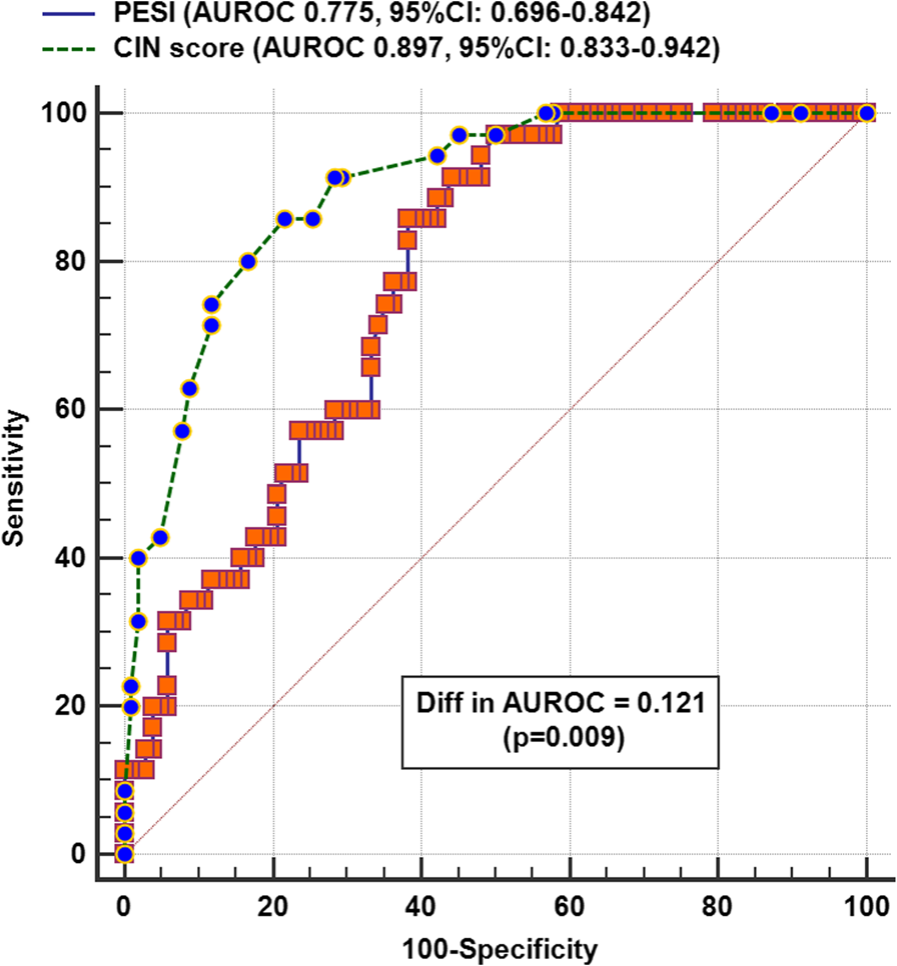
Areas under the receiver-operating-characteristic (AUROC) curve of the Pulmonary Embolism Severity Index (PESI) and contrast-induced nephropathy (CIN) prediction score to predict risk of requiring dialysis

Similarly, the CIN prediction score was also better than the PESI in discriminating between patients who required subsequent dialysis and those who did not (AUROC 0.897, 95% CI 0.833–0.942 vs. 0.775, 95% CI 0.696–0.842, respectively; difference in AUROC 0.121, *p* = 0.009) (Fig. 3).

**Fig. 4:**
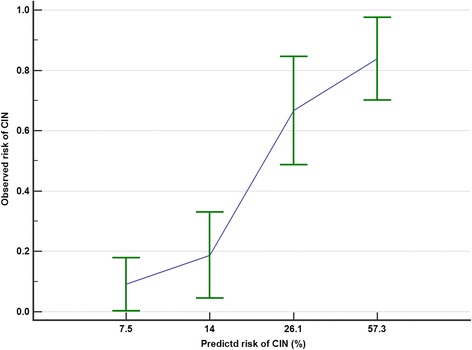
The relationship between the contrast-induced nephropathy (CIN) prediction score’s predicted and observed risks of CIN

**Fig. 5:**
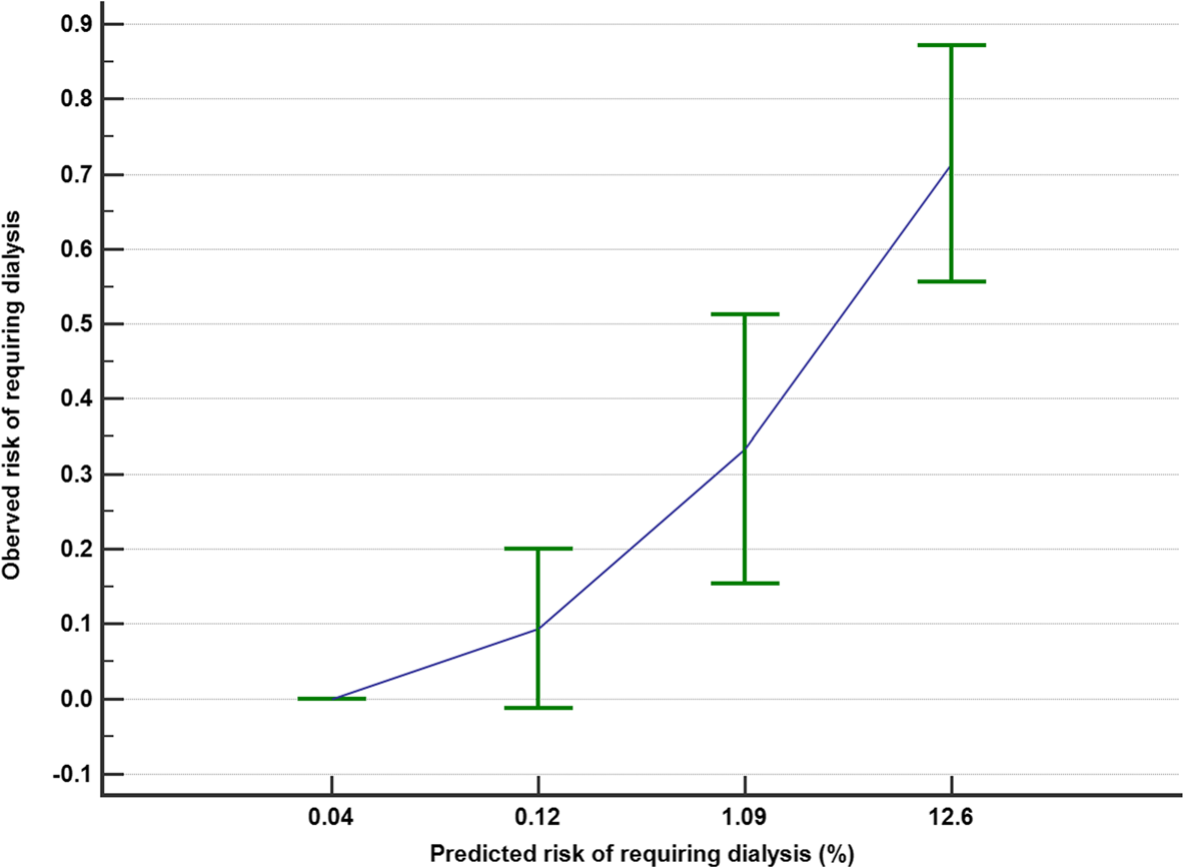
The relationship between the contrast-induced nephropathy (CIN) prediction score’s predicted and observed risks of dialysis

**Fig. 6:**
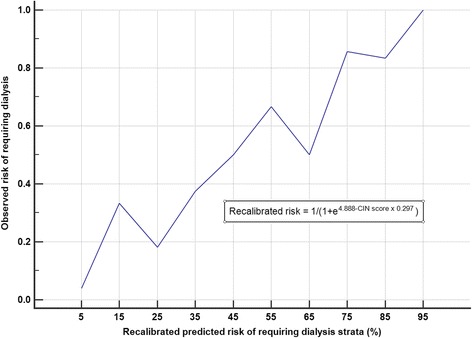
The relationship between the contrast-induced nephropathy (CIN) prediction score’s predicted and observed risks of requiring dialysis after recalibrating the prediction equation’s slope and intercept

The calibration plots showed that the CIN prediction score underestimated both the observed risks of CIN (Hosmer-Lemeshow Chi-square = 9.4, *p* = 0.009) and dialysis (Hosmer-Lemeshow Chi-square = 15.3, *p* = 0.001) (Figs. 4 and 5). After recalibration of the slope and intercept of the prediction equation, the CIN predicted risks of requiring dialysis after a CTPA were more closely related to the observed risks of dialysis (recalibrated CIN risk = 1/(1 + e^4.888−CIN score × 0.297^) (Fig. 6).

**Fig. 7:**
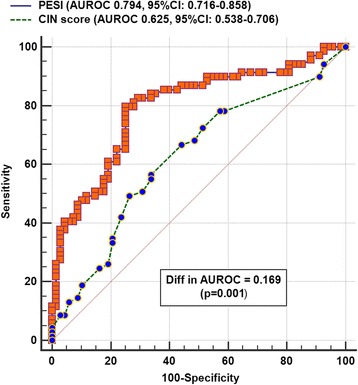
Areas under the receiver-operating-characteristic (AUROC) curve of the Pulmonary Embolism Severity Index (PESI) and contrast-induced nephropathy (CIN) prediction score to predict risk of hospital mortality

Despite not all study patients had a confirmed acute PE, the PESI still had a satisfactory ability and indeed far better ability than the CIN prediction score to discriminate between survivors and non-survivors (AUROC 0.794, 95% CI 0.716–0.858 vs. 0.625, 95% CI 0.538–0.706; difference in AUROC 0.169, *p* = 0.001) (Fig. 7). CIN prediction score and PESI were, however, both unsatisfactory in predicting the presence of PE on the CTPA (both AUROC < 0.6).

## Discussion

This study showed that CIN (41%) and dialysis (26%) after a CTPA scan were not rare in the critically ill with a suspected life-threatening acute PE. In patients with unstable vital signs and physiology requiring multiple organ support, the CIN prediction score had a good ability to discriminate between patients with and without developing CIN (AUROC 0.864) and requiring dialysis (AUROC 0.897) subsequently and was better than the PESI in predicting both outcomes. A CIN risk score > 10 and 12 had an 82.1 and 85.7% sensitivity and 81.5 and 78.4% specificity to predict subsequent CIN and dialysis, respectively. However, the CIN prediction score tended to underestimate the risks of CIN and dialysis and was also inferior to the PESI in predicting mortality in the critically ill with suspected PE. These results have some clinical implications and require further discussion.

First, despite an increasing awareness of the importance of VTE in the critically ill, PE remains as one of the frequently missed diagnoses—identified only in autopsies [20–23]. Because the pre-test probability of VTE in the critically ill is high, d-dimers are not useful and CTPA has practically become the gold standard to confirm or exclude a life-threatening acute PE [20]. This practice is further supported by the fact that a CTPA can also evaluate the right ventricular size, which reflects the severity of acute PE. In addition, a CTPA may provide important information on alternative differential diagnoses such as pneumonia. Nevertheless, our results suggest that there is a risk of CIN associated with any radiocontrast studies, including a CTPA scan, in the critically ill. It is possible that the deteriorations in renal function after a CTPA in some of our patients could occur regardless of whether radiocontrast had been used, or at least in part, if not purely due to the natural progression of a life-threatening illness. The differential ability of the CIN prediction score and PESI in predicting CIN and mortality suggests that the severity of illness alone may not fully explain the risks of renal dysfunction and dialysis after a CTPA scan. PESI and CIN prediction score do share some similar risk factors (such as age, congestive heart failure, and hypotension), but there are also factors in the CIN prediction score that are distinct from the PESI, including diabetes mellitus, anaemia, and pre-existing renal impairment [10, 18, 19]. As such, we can argue that radiocontrast was likely to play a contributing role in inducing the increases in plasma creatinine concentrations in our patients, especially in those with multiple risk factors for CIN [10, 18, 19].

**Fig. 8:**
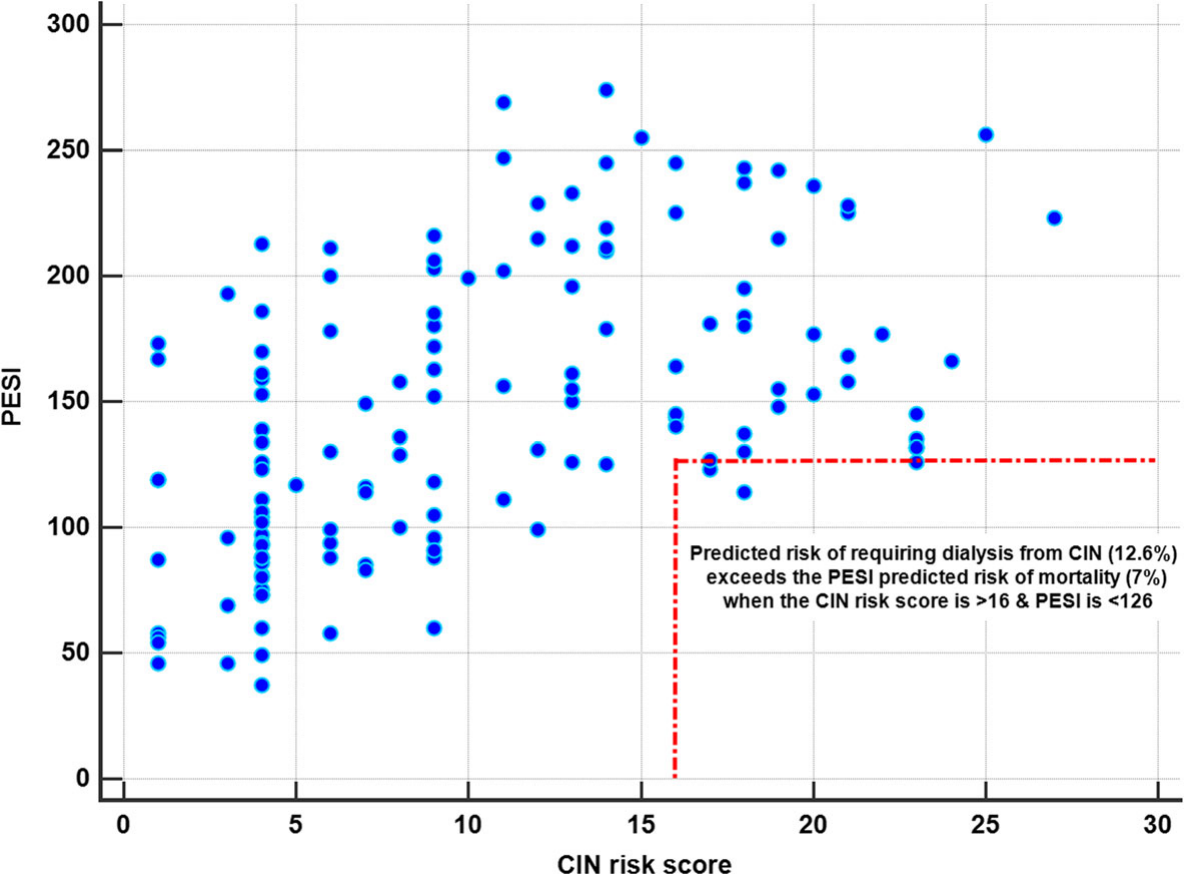
The situations when contrast-induced nephropathy (CIN) prediction score’s predicted risk of dialysis exceeds the Pulmonary Embolism Severity Index (PESI)’s predicted risk of mortality

Our results also suggest that the CIN prediction score and PESI may also have complementary roles in determining the balance between benefits and harms of a CTPA in a patient with suspected acute PE. Theoretically, a CTPA will be justifiable if the predicted risk of mortality by the PESI is higher than the predicted risk of requiring dialysis by the CIN prediction score in any patients with symptoms and signs of acute PE. Conversely, if the CIN prediction score’s predicted risk of requiring dialysis is much higher than the predicted risk of mortality by the PESI (when the CIN risk score is > 16 and PESI is < 126) (Fig. 8), perhaps an alternative way to diagnose or exclude an acute PE, possibly by a combination of tests—such as transoesophageal echocardiography, lower limb venous ultrasonography, and ventilation-perfusion scan—should be seriously considered in reducing the long-term consequences of CIN [10, 24]. However, a delay in doing a CTPA scan to allow aggressive intravenous hydration or other forms of CIN prophylaxis does not confer substantial benefits in reducing CIN after CTPA in an acute care setting [25, 26].

Second, our study showed that the CIN prediction score had a good discriminative ability but was not well-calibrated in predicting both CIN and dialysis after a CTPA in the critically ill. It should be noted that our primary results—AUROC, sensitivity, and specificity—are known to be not affected by prevalence (or pre-test probability) of the outcome of interest. In addition, after recalibration of the intercept and slope of the existing CIN prediction equation, the predicted risks of requiring dialysis appeared to match the observed risks much better (Fig. 7). This result suggests that the covariates used in the CIN prediction are largely ‘good or valid’ predictors for CIN in the critically ill, even though they are derived from cardiology patients. Hence, further refinement and recalibration of the CIN prediction score for patients who require a CTPA will be feasible, and relatively straightforward, by a prospective multicentre cohort study. Using a conservative rule of one covariate per ten outcomes and assuming an incidence (or pre-test probability) of CIN is 20% (or 10%), a sample size > 600 (or 1200) patients will be needed to develop a CIN prediction model with 12 predictors for the critically ill which can then be validated with a similar or larger size study.

Finally, we need to acknowledge the limitations of this study. Although the CIN prediction score has included a number of risk factors for renal dysfunction after administration of radiocontrast, there are other risk factors that might also be important, including use of nephrotoxic drugs and presence of infection. The significance of these risk factors in predicting CIN should be considered in developing a CIN prediction model for patients undergoing a CTPA scan. With a relatively small sample size, we were unable to test the relative importance of each risk factor contained in the CIN prediction score, both in predicting CIN as well as the presence of PE and its associated mortality. As such, a large prospective multicentre study will be essential to improve our understanding on when, and in whom, a CTPA will be most beneficial.

## Conclusions

In summary, CIN and dialysis were not rare in critically ill patients who had unstable vital signs after using CTPA scan to exclude a life-threatening acute PE. Despite only derived from cardiology patients, the CIN prediction score had a good ability to discriminate between patients with and without developing CIN and requiring dialysis after an urgent CTPA scan and indeed was better than the PESI in predicting both adverse renal outcomes. Further research is, however, needed to improve the calibration of the current CIN prediction score or reconstruct an even more accurate prediction score in predicting CIN and dialysis in the critically ill who require different types of radiocontrast study. Used together for critically ill patients with suspected acute PE, the CIN prediction score and PESI may be useful to inform clinicians when the benefits of a CTPA scan will outweigh its potential harms.

## Additional file

Additional file 1: Table S1. TRIPOD Checklist. (DOCX 17 kb)

## Abbreviations

AUROC: Area under the receiver-operating-characteristic
CIN: Contrast-induced nephropathy
CTPA: Computed tomography pulmonary angiography
PE: Pulmonary embolism
PESI: Pulmonary Embolism Severity Index
